# Supplementary material for: Dental and Dental Hygiene Students’ Knowledge and Capacity to Discriminate the Developmental Defects of Enamel: A Self-Submitted Questionnaire Survey
Source: Children (Basel). 2022 Nov 16;9(11):1759. doi: 10.3390/children9111759 (PMC9688626; doi:10.3390/children9111759)
Supplement: Supplementary file 1 [file children-09-01759-s001.zip › Supplementary file S1.pdf]

Category	Questions	Type of answers
**Demographics**	1. Gender	Multiple-choice answers
	2. Year and type of study	Multiple-choice answers
	3. In which area do you attend the Degree Course?	Multiple-choice answers
**Basic knowledge**	4. Are you receiving or have you received information regarding DDEs (Developmental defect of enamel)?	Yes/no
	5. Where did you get information about DDE?	Multiple-choice answers
	6. Which type of defects is enamel hypomineralization	Multiple-choice answers
	7. Which type of defects is hypoplasia	Multiple-choice answers
	8. The Molar incisor hypomineralization (MIH) is a defect	Multiple-choice answers
	9. In which phase of dental development Molar Incisor hypomineralization starts	Multiple-choice answers
	10. Amelogenesis Imperfecta is a condition caused by	Multiple-choice answers
	11. In which phase of dental development Amologenesis Imperfecta starts	Multiple-choice answers
	12. In which phase of dental development fluorosis starts:	Multiple choice answers
	13. In your opinion, fluorosis can be easily confused with the demineralization effect of dental plaque (white spot/brown spot)?	Yes/No
	14. How confident are you in your ability, to clinically detect DDE and other diseases that affect hard tissue?	Likert scale
**Clinical picture**	15. CASE 1: How would you define this condition?	Multiple-choice answers
	16. In your opinion, what kind of caries risk does the subject present?	Likert scale
	17. Which of the following treatment would you recommend? (More answers possible)	Multiple-choice answers
	18. CASE 2: How would you define this condition?	Multiple-choice answers
	19. In your opinion, what kind of caries risk does the subject present?	Likert scale
	20. Which of the following treatment would you recommend? (More answers possible)	Multiple-choice answers
	21. CASE 3: How would you define this condition?	Multiple-choice answers
	22. In your opinion, what kind of caries risk does the subject present?	Likert scale
	23. Which of the following treatment would you recommend? (More answers possible)	Multiple-choice answers
	24. CASE 4: How would you define this condition?	Multiple-choice answers
	25. In your opinion, what kind of caries risk does the subject present?	Likert scale
	26. Which of the following treatment would you recommend? (More answers possible)	Multiple-choice answers
	27. CASE 5: How would you define this condition?	Multiple-choice answers
	28. Which of the following treatment would you recommend? (More answers possible)	Multiple-choice answers

Supplementary Table S1: Questionnaire items and type of answers
